# Performance Evaluation of Thermal Insulation Materials from Sheep’s Wool and Hemp Fibres

**DOI:** 10.3390/ma17133339

**Published:** 2024-07-05

**Authors:** Sigitas Vėjelis, Saulius Vaitkus, Virgilijus Skulskis, Arūnas Kremensas, Agnė Kairytė

**Affiliations:** 1 Building Materials Institute, Faculty of Civil Engineering, Vilnius Gediminas Technical University, Linkmenų str. 28, LT-08217 Vilnius, Lithuania; 2 Institute of Economics and Rural Development, Lithuanian Centre for Social Sciences, 03220 Vilnius, Lithuania; virgilijus.skulskis@ekvi.lt

**Keywords:** natural fibres, thermal insulation materials, industrial hemp fibres, sheep wool, polylactide fibres, performance

## Abstract

In the current work, the performance properties of natural-fibre-based thermal insulation materials were examined. For this purpose, three different compositions of natural fibres were prepared: pure sheep wool (SW), wool and industrial hemp (SW/HF) fibres, and pure industrial hemp (HF) fibres. Low-melt bicomponent polylactide (PLA) fibres were used as a binding material. For specimens prepared from natural fibres, the dependence of the thermal conductivity, the tensile strength along and across the direction of product formation, and the short-term water absorption on the density of the specimens and the flammability parameters were determined. In addition, to reduce the water absorption and flammability, the specimens were coated with hydrophobic agents and flame retardants. The obtained research results were also statistically processed. The analysis of the results showed that the thermal conductivity of natural-fibre-based thermal insulation materials varied within the range of 0.0333 ÷ 0.0438 W/(m·K), the tensile strength varied from 2.5 to 130 kPa, the short-term water absorption varied from 0.5 to 8.5 kg/m^2^, and the water vapour diffusion resistance factor varied from 2.537 to 2.667. It was additionally determined that all the studied products were flammable. The water absorption and flammability values were significantly reduced by the use of hydrophobic agents and flame retardants.

## 1. Introduction

The construction sector is one of the primary industry sectors causing a negative environmental impact [1]. The choice of a thermal insulation layer not only determines the thermal resistance of a building and its microclimate but also the impact on the environment and human health [2,3]. Research suggests that there is a need to develop insulating materials that are relatively cheap, possess excellent properties, and, at the same time, have a low negative environmental impact [4,5]. Most thermal insulation materials are still produced from fossil-based raw materials. Approximately 90% of thermal insulation materials consist of fibrous mineral and polymer foam materials [6,7].

In the last two decades, natural fibres from cultivated and wild agricultural plants, the wood-processing industry, animal wool, etc., have received much attention as alternatives for thermal insulation applications due to their widespread availability, sustainability, and cost-effectiveness [8,9,10]. The use of harmful formaldehyde-based binders has also been banned in binding natural fibres. They are usually replaced by more environmentally friendly alternatives such as biopolyurethane [11], liquid sodium silicate [12], binders synthesised from various plants [13], or self-binding materials [14].

Thermal insulation materials produced from renewable resources are not only a lever for materials produced from petroleum products, but they also have several advantages. These advantages include their biodegradability, low thermal conductivity, sufficient acoustic properties, and low-resource production techniques [15,16].

The main indicator of thermal insulation materials that allows one to compare the thermal insulation efficiency of different materials is thermal conductivity [17]. According to the standards of thermal insulation materials [18,19,20,21], materials with a thermal conductivity of no more than 0.06 W/(m·K) are classified as thermal insulation materials. The thermal conductivity of most classical thermal insulation materials varies between 0.030 and 0.046 W/(m·K) [22,23]. When new products are developed, it is important to achieve a similar thermal conductivity value to maintain their competitiveness in the market. Other performance characteristics, such as the short-term and long-term compressive strength, the flexural strength, the dimensional stability, the long-term water absorption, etc., of the products are important, but they are not always determined because they depend on the product’s application area and exploitation conditions.

In the scientific literature, there are many examples of the development of thermal insulation materials from natural fibres [24]. Fibres are used for the production of thermal insulation composites, thermal insulation mats, and boards. Much attention is being paid to industrial hemp fibres, because the hemp plant produces the largest dry raw material mass per year [25].

Lekavicius et al. [26] analysed 13 different thermal insulation materials, the main component of which was hemp fibres. The density of these thermal insulation materials varied from 25 to 42 kg/m^3^, and the thermal conductivity varied from 0.038 to 0.043 W/(m·K). Additionally, the thermal insulation materials analysed by the researchers [26] were bonded with natural and synthetic binders with the incorporation of various additives to improve the properties of the materials.

In [27], thermal insulation composites were made from industrial hemp fibres and polyurethane as a binding material. The content of hemp fibres ranged from 0 to 30%. The authors found that increasing the amount of hemp fibres increased the thermal conductivity from 0.031 W/(m·K) when no hemp fibres were used to 0.041 W/(m·K) when 30% hemp fibres were added. The hemp fibres also played an important role in the variation in tensile strength. When no hemp fibres were used, the tensile strength was 1.03 MPa, and the tensile strength increased with the addition of small amounts of hemp fibre. The highest tensile strength was achieved when 15% hemp fibre was used. As the amount of hemp fibres increased further, the tensile strength decreased, and when 30% hemp fibre was used, the tensile strength was only 0.675 MPa. A similar trend was observed in the bending strength tests. The maximum bending strength was 3.83 MPa with 20% fibre and 2.61 MPa with 30% fibre.

In another work [28], the researchers examined a large number of products made from flax and hemp fibres. The density of these products ranged from 5 to 100 kg/m^3^, and the thermal conductivity ranged from 0.035 to 0.094 W/(m·K). Scientists note that there is no linear relationship between the fibre density and the thermal conductivity of materials derived from flax and hemp. Therefore, the research results obtained by different scientists differ significantly and are determined by a series of circumstances. First, these properties are related to the morphological structure and chemical composition of the developed products, which in turn are dependent on the growth conditions; stem-soaking duration; special separation techniques based on mechanical, biochemical, and microbiological methods; product density and thickness; air permeability; humidity; temperature; etc.

Another group of researchers [29] investigated the hygric properties, namely the adsorption–desorption isotherm, the moisture buffer value, the water vapour diffusion resistance factor, and the water absorption coefficient. They tested commercial hemp-fibre-based thermal insulation products with a density of 39–60 kg/m^3^ and a thermal conductivity of 0.038–0.043 W/(m·K). The scientists found that the properties of almost all the products were very different; therefore, they concluded that the processing of fibres, the fibrous structure, the tortuosity, and the porosity of the insulation materials influence their moisture-sorption and moisture-diffusion properties.

In [30], researchers examined the flammability of thermal insulation materials developed from differently processed hemp fibres. Nonwoven samples were made using needle-punching, thermal bonding, and hydro-entangling. The density of the products ranged from 24.3 to 114.5 kg/m^3^, and the thickness from 3.3 to 17.7 mm. Scientists determined the influence of fibre variety, density, fibre origin, and material production technology on flammability. The researchers note that further research on flame retardant finishing should be performed to improve protection against combustion and hinder flame spread.

Among the fibres of animal origin, sheep’s wool is mainly used. Scientists [31] studied the impact of the density of sheep wool boards on thermal conductivity within vast limits. In this case, the density of the wool boards ranged from 35 to 248 kg/m^3^, while the thermal conductivity varied from 0.0324 to 0.0436 W/(m·K). The samples were prepared with two different binding materials—latex and resin. The samples bonded with latex were characterized by a lower thermal conductivity ranging from 0.0324 to 0.0361 W/(m·K) at a 35 to 185 kg/m^3^ density range. The authors also found that when latex is used as the binding material, the water absorption does not exceed 0.94 kg/m^2^, while the maximum value reaches even 5.52 kg/m^2^ when the resin is added. Furthermore, the use of latex results in a significantly better water vapour permeability. The water vapour diffusion resistance factors of the samples bonded with latex and resin are 1.6 and 2.9, respectively.

In another work [32], researchers developed one thermal insulation material from pure wool and another using 50% wool and 50% hemp fibres. The density of the materials ranged from 21 to 142 kg/m^3^, and the thermal conductivity from 0.038 to 0.041 W/(m·K). The authors note that mixing hemp fibres into sheep’s wool does not affect thermal conductivity and contributes to a decrease in the environmental impact while at the same time assuring a higher stiffness and easy installation.

The literature analysis allows us to conclude that thermal insulation materials produced from fossil resources can often be replaced by thermal insulation materials produced from natural fibres. Insulation made from natural resources is not competitive compared to classical thermal insulation materials. To produce competitive thermal insulation materials from natural fibres, it is necessary to strictly control the initial raw materials at various stages of their preparation and their processing processes, properly select their composition and additives that improve properties, select the appropriate production technology, and control the density of the materials to ensure their performance characteristics.

The purpose of this work is to consistently examine the impact of the density of thermal insulation materials from SW, HF, and SW/HF fibres on thermal conductivity, tensile strength along and across the formation direction, hygric parameters, and flammability characteristics. The novelty of this work is a detailed comparison of the performance characteristics of thermal insulation materials made from two different fibres and their mixture. Knowledge of the influence of different fibres on the performance properties of thermal insulation materials is necessary for the development and production of ecological thermal insulation materials.

## 2. Materials and Methods

### 2.1. Raw Materials

Three compositions of thermal insulation materials were used for the research. The compositions are presented in Table 1.

PLA fibres (PLA Ingeo™) were obtained from Max Model S.A.S (1 Quai Jules Courmont, Lyon, France). PLA was chosen because it is obtained by synthesizing natural materials. The main characteristics of the PLA fibres were as follows: 4.4 dtex, length—51 mm, and melting temperature—130 °C. SW was obtained from the Lithuanian farm (Impoliai 2, Pakruojis, Lithuania). In the current research, wool of the German Merinolandschaf sheep breed was used. The wool was washed, and the remaining fat content was less than 1%. The diameter of the wool fibres varied from 22 to 28 μm, length 70–90 mm.

Industrial HF fibres were obtained from UAB Natūralus Pluoštas (Biochemiku st. 5, Kėdainiai, Lithuania). Fibres were extracted from the Austa SK industrial hemp variety. The length of the fibres was 40–60 mm, and the diameter of the fibres was 17–22 μm.

**Figure 1:**
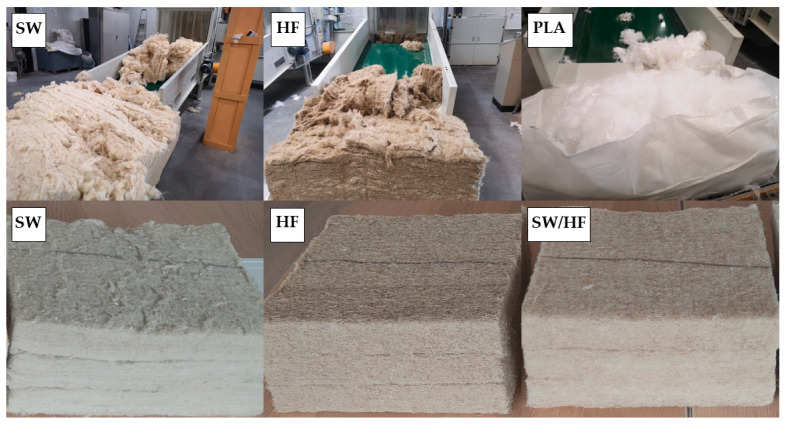
A general view of different fibres and prepared specimens.

Figure 1 presents a general view of the SW, HF, and PLA fibres prepared for the production of samples and samples with different compositions.

### 2.2. Preparation of Thermal Insulation Materials

Using carding technology, thermal insulation materials were produced at the textile company UAB Neaustima (J. Basanavičiaus st. 103C, Šiauliai, Lithuania). Before carding, the SW was pre-cut to 4–8 cm. PLA and natural fibres were mixed using an internal mixer. The mixed fibres were combed in the same direction during the carding process to create composite webs. Eighteen webs were laid on each other to obtain a 50 mm-thick product. The folded breams were fed into the curing oven and heated at 160 °C for 3 min. After thermal treatment, the web was compressed between two drums to obtain the required density and thickness of the material. The thermally treated and cooled thermal insulation materials were cut to the required length and width.

### 2.3. Testing Methods

Test specimens with dimensions of (300 × 300) mm and thickness of (50 ± 5) mm were prepared for the thermal conductivity measurements. The thermal conductivity test was performed using the constant heat flow method according to the requirements of EN 12667 [33]. The thermal conductivity was determined at an average temperature of 10 °C. Before the test, all specimens were conditioned for 72 h at a temperature of (23 ± 2) °C and a relative air humidity of (50 ± 5)%. Three specimens of each density and each type of thermal insulation were prepared.

The tensile strength of the specimens parallel to the surfaces was determined according to the methodology specified in the ISO 29765 [34] standard. The test directions for the tensile tests were across and along the direction of product formation. Before the test, all specimens were conditioned for 24 h at a temperature of (23 ± 2) °C and a relative air humidity of (50 ± 5)%. Three specimens were prepared for each type of product.

Short-term water absorption was determined according to method A of EN ISO 29767 [35]. The test direction for short-term absorption was perpendicular to the surface of the specimen. The specimens were loaded with a sufficient load to immerse them when water was added partially. The bottom surface of the specimen was (10 ± 2) mm below the surface of the water during the test. After immersion in water for 24 h, the specimens were removed and weighed, and the short-term absorption was calculated. Three samples were prepared for each type and density of the product. Before the test, all specimens were conditioned for 24 h at a temperature of (23 ± 2) °C and a relative air humidity of (50 ± 5)%. The hydrophobic agent Beiphob FR (CHT Germany GmbH, Tübingen, Germany) was applied to reduce water absorption of thermal insulation materials. This hydrophobic agent is produced based on a fluoroalkyl acrylate copolymer. A water repellent was sprayed on the fibres before the mat was formed.

The water vapour diffusion resistance factor of the specimens was determined according to ISO 12629 [36]. The permeability of the water vapour was determined under dry climatic conditions at 23 °C and 0/50%. Three (100 × 100 × 100) mm specimens of each composition and density were tested. Before the test, the specimens were conditioned for 6 h at a temperature of (23 ± 5) °C. The average air pressure during the test was 1009 hPa. The flow direction of the water vapour stream was perpendicular to the forming surface of the products.

For the flammability test, the products were exposed to an open flame according to the requirements of EN ISO 11925-2 [37]. The method to determine flammability is based on the direct exposure of vertical samples to a small flame in the presence of zero external energy illumination. During the test, information was recorded on whether the specimen ignites, how the flame spreads above the flame point, and the fact of smouldering. Three specimens were prepared for each type of product. The dimensions of the specimens were (200 × 90 × 50) mm. The specimens were exposed to an open flame at an angle of 45° for 15 s. After removing the flame source, the specimens were left to burn for 5 s. To reduce the flammability of thermal insulation, the flame retardant Apyrol BKW (CHT Germany GmbH, Tübingen, Germany) was applied. Apyrol BKW is a commercially available phosphorus and sulphur compound. Two main conditions were considered when choosing a flame retardant. They had to be applied by spraying and have a minimal environmental impact. Flame retardant was sprayed on the fibres before the mat was formed. Previous studies [38] used the Flovan CGN agent to protect the thermal insulation material from HF when a solution concentration of 45 g/L was used. In the current study, a solution concentration of 45 g/L was chosen for all types of thermal insulation developed.

For processing the experimental data and evaluating their reliability, mathematical-statistical methods and the software STATISTICA v.8 were used [39].

## 3. Results and Discussions

### 3.1. Thermal Conductivity

**Figure 2:**
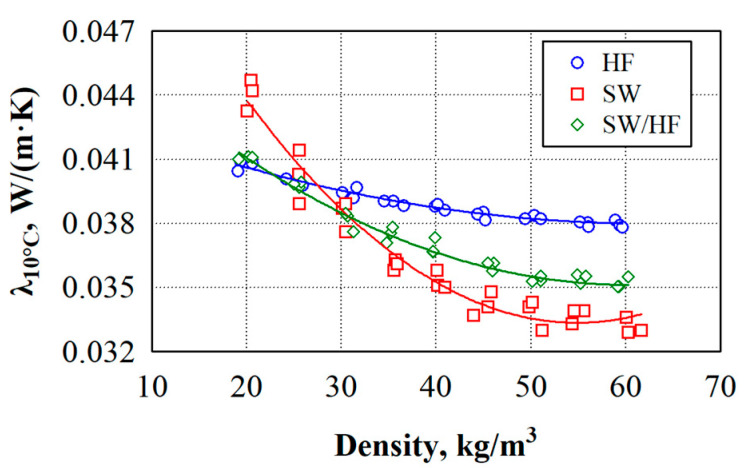
Dependence of thermal conductivity on density of thermal insulation materials: (—)—average values according to the regression equation.

Figure 2 shows that the density of the specimens prepared for the thermal conductivity tests varied from 20 to 60 kg/m^3^. The specimens with the lowest density had the highest thermal conductivity, and those with a density of 50–60 kg/m^3^ had the lowest thermal conductivity value. The change in thermal conductivity with the varying density is related to the heat transfer methods in the product itself [40,41]. When the density of the material is low, heat is intensively transferred through the air. As the density increases, the heat transfer through the air decreases because the air gaps in the material decrease, and when a specific density of the material is reached, the heat transfer through the solid framework of the material begins to intensify. In some cases [42], when the fibres are coarse, the air gaps decrease more slowly, so more intense heat transfer through the solid frame of the materials does not start or starts with a higher density of the thermal insulating material.

The analysis of the experimental results shows a strong dependence of the thermal conductivity values of the developed thermal insulation materials on their density (see Figure 2).

Therefore, linear regression equations can describe the thermal conductivity values of such thermal insulation materials (see Table 2).

Using various models, researchers [41] have found that thermal insulation materials from renewable resources with a density of 50–90 kg/m^3^ have the lowest thermal conductivity, i.e., 0.0310–0.0330 W/(m·K). In the current case, the SW thermal insulation material has the lowest thermal conductivity when the density is 50–60 kg/m^3^, and the thermal conductivity varies from 0.0333 to 0.0335 W/(m·K). Furthermore, researchers [41] have investigated natural fibre-based insulating materials with fibre diameters ranging from 5 to 50 μm. The authors note that reducing the diameter of the fibres to form nanometer-sized pores is an effective way to improve the thermal insulation of such materials. The thickness of the fibres used in the current work is 17–28 μm. The absence of small-diameter fibres in the thermal insulation material likely prevents the achievement of a lower thermal conductivity value. It should also be noted that, although HF is slightly thinner than SW, HF is rougher than SW; therefore, it is more difficult to compress, and more intensive heat transfer through the solid framework of materials does not occur when the thermal insulation material is produced from HF and SW/HF.

### 3.2. Tensile Strength

**Figure 3:**
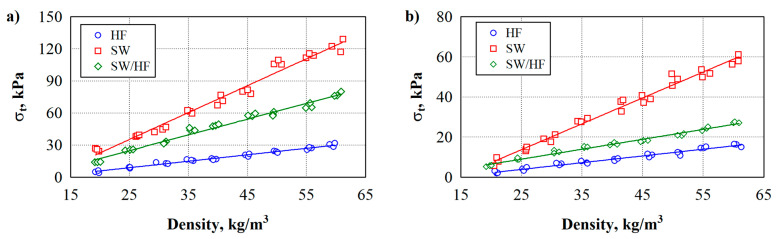
Experimental values of the tensile strength of natural thermal insulation materials: (**a**) across the forming direction; (**b**) along the forming direction.

Figure 3 shows the tensile strength results. Analysis of the experimental results shows a strong dependence of the tensile stress σ_t_ of the developed materials on their density across and along the direction of the formation (see Figure 3). Therefore, the linear regression equations can describe the tensile strength values of thermal insulation materials (see Table 3).

As the density of the fibrous composite specimens increases from 20 to 60 kg/m^3^, the tensile strength of the transverse fibres increases linearly. The increase for specimens made from SW is 405%, HF—439%, and SW/HF—335%. Tensile strength along the fibres also increases linearly. The increase for specimens made from SW is 599%, HF—707%, and SW/HF—302%.

The analysis of the experimental data shows that the lowest tensile strength is observed for SW specimens made across and along the forming direction, while the highest is for HF specimens.

Comparing tensile strength values between fibres oriented across and along the forming direction of thermal insulation materials, the difference between specimens at a density of 20 kg/m^3^ is SW—166%, HF—213%, and SW/HF—168%. Comparing tensile strength values between fibres oriented across and along the forming direction of thermal insulation materials, the difference between specimens at a density of 60 kg/m^3^ is SW—92%, HF—109%, and SW/HF—190%. Thermal insulation materials have the highest tensile strength when their forming direction is across-oriented.

Researchers [43] have studied the strength variation in different fibres. The authors note that the tensile strength of HF reaches 310–900 MPa, while for SW, it is only 120–174 MPa. In the current case, the maximum tensile strength of the developed thermal insulation materials reaches 130 kPa. It shows that the formation of contact zones between natural fibres and PLA has the most significant influence on the tensile strength. In the work in [44], it is observed that weak contact zones are usually formed due to the nature of the materials and their different polarity. Furthermore, the adhesion of SW fibres to PLA is likely poorer due to the grease remaining in the wool after washing. However, despite the formation of sufficiently weak contact zones, the strength of thermal insulation materials when stretched along and across the direction of formation is sufficient as it easily meets the requirement to withstand double the product’s weight.

### 3.3. Short-Term Water Absorption

**Figure 4:**
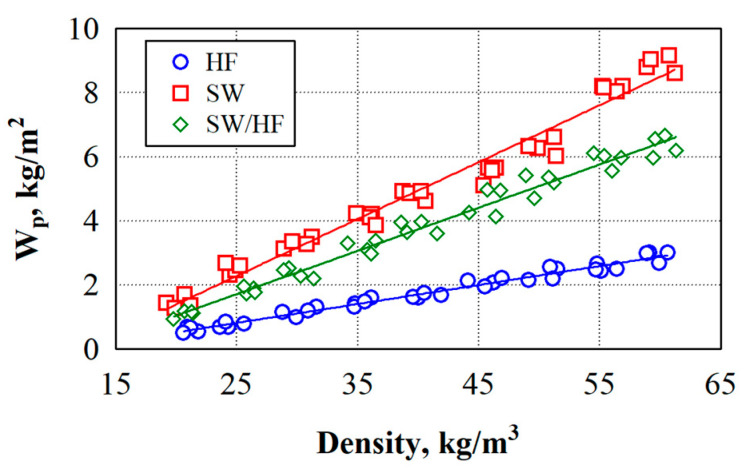
Experimental values of the short-term water absorption of natural thermal insulation materials.

Figure 4 shows the results of the short-term water absorption tests. After analysing the experimental data, the dependence of the thermal insulation materials’ short-term water absorption W_p_ on the density becomes apparent (see Figure 4). Therefore, the linear regression equations can describe the short-term water absorption values of thermal insulation materials (see Table 4).

As the density of the specimens increases from 20 to 60 kg/m^3^, the short-term water absorption linearly increases for specimens made from SW by 452%, HF by 515%, and SF/HF by 508%. Additionally, at a density of 60 kg/m^3^, it reaches 2.85, 6.50 and 8.8 kg/m^2^, respectively. 

Analysis of the experimental data shows that SW specimens have the lowest water absorption and growth, while the HF specimens have the highest. At a thermal insulation density of 20 kg/m^3^, the difference in short-term water absorption between SW and HF specimens is 2.7 times; between SW and SW/HF specimens, it is 2.0 times; and between HF and SW/HF specimens, it is 1.3 times. At a density of 60 kg/m^3^, the difference in short-term water absorption between SW and HF specimens is 2.9 times; between SW and SW/HF specimens, it is 2.2 times; and between HF and SW/HF specimens, it is 1.3 times. The lowest difference in short-term water absorption is observed between HF and SW/HF specimens, while the largest is obtained for specimens made from HF and SW. The small amount of fat on the wool fibres likely acts as a water-repellent, so as the density of the thermal insulation material made of SW increases, the absorption intensity is significantly lower than that of the material made of HF.

**Figure 5:**
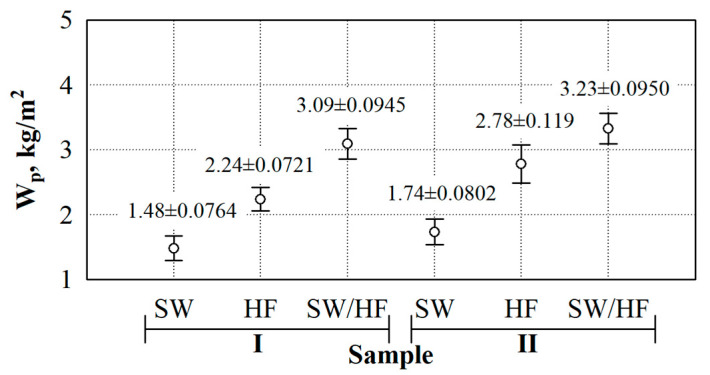
Short-term water absorption of thermal insulation materials coated with additives: Sample I—with hydrophobic agent; Sample II—with hydrophobic agent and flame retardant.

Additional short-term water absorption tests were performed using thermal insulation materials with a 40 kg/m^3^ nominal density. This density was chosen as the average density of the studied thermal insulation materials. In this case, specimens were coated with hydrophobic agents and flame retardants. The actual density values of the specimens coated with hydrophobic agents are as follows (see Figure 5, Sample I): SW—41.1 ± 0.305 kg/m^3^, HF—41.3 ± 0.472 kg/m^3^, and SW/HF—41.2 ± 0.361 kg/m^3^. The actual density values of the specimens coated with hydrophobic agents and flame retardants are as follows (see Figure 4, Sample II): SW—41.2 ± 0.265 kg/m^3^, HF—40.3 ± 0.624 kg/m^3^, and SW/HF—41.1 ± 0.252 kg/m^3^.

Repeated analysis of the short-term absorption tests of specimens coated with various additives shows that the average values of different specimens differ (see Figure 5). The statistic of the F-criterion is 201.5, and *p* < 0. This indicates a statistically significant difference in the results of the subject. Consequently, the coefficient of determination R^2^ = (0.988) and the adjusted coefficient of determination R^2^ = (0.983) are obtained.

It can be seen that the short-term water absorption decreased after the specimens were coated with a hydrophobic agent. The short-term water absorption value decreased by ~20% for uncoated and coated SW specimens with a 41.1 kg/m^3^ density. Moreover, the parameter decreased by 75% for HF specimens at a 41.3 kg/m^3^ density. Lastly, it was reduced by 67% for SW/HF specimens at a 41.2 kg/m^3^ density.

Short-term water absorption was reduced in all cases after the specimens were coated with a hydrophobic agent and a flame retardant. For SW specimens with a density of 41.2 kg/m^3^, the parameter decreased by 2.3%. Additionally, for HF specimens at a density of 41.3 kg/m^3^, it decreased by 36%; for SW/HF specimens at a density of 41.2 kg/m^3^, it was reduced by 59%.

Depending on the type of fibres, short-term water absorption can be reduced from 20 to 75% for hydrophobic agent-coated specimens. In addition, specimens coated with a hydrophobic agent and a flame retardant exhibited short-term water absorption values reduced from 2.3 to 59%. It can be distinguished that applying the flame retardant to the already hydrophobized specimens also increases the parameter.

In the works of other authors [45,46], it is noted that SW fibres absorb moisture from the air and, once immersed in water, take up considerable amounts of liquid. Scientists [47] have also used SW to develop thermal insulation materials. They found that after coating SW with hydrophobic agents, the wettability of the thermal insulation material was reduced by up to 228%. In the current case, our findings inspire new possibilities, as we have managed to reduce the absorption by up to 75% when the specimens were prepared from HF and by only 20% when the specimens were prepared from SW.

### 3.4. Water Vapour Permeability

**Figure 6:**
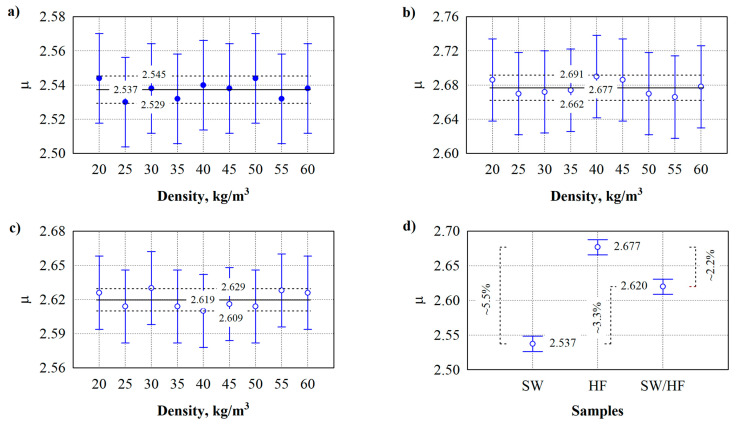
Test results of the water vapour resistance factor of thermal insulation materials made from (**a**) SW; (**b**) HF; and (**c**) SW/HF. (**d**) Comparison of the water vapour resistance factor of the thermal insulation materials.

Figure 6 shows studies of the water vapour permeability of thermal insulation materials. After conducting research, it is found that the density of different types of thermal insulation materials does not significantly affect water vapour permeability (see Figure 6a–c). However, the difference is noticeable when comparing different thermal insulation materials (see Figure 6d). Table 5 lists the developed thermal insulation materials’ accepted nominal and actual densities for water vapour permeability tests.

The water vapour permeability analysis of the specimens shows that when the density of the material varies from 20 kg/m^3^ to 60 kg/m^3^, the average values of the water vapour resistance factor µ for different specimens do not differ (see Table 6). The statistic of the F-criterion is 0.13 ÷ 0.23, and *p* > 0.98. This indicates a statistically insignificant difference in the results of the subject. Consequently, the determination coefficient R^2^ = (0.028 ÷ 0.049) and the adjusted determination coefficient R^2^ = (−0.16÷−0.19) are obtained. Thus, when the density of the specimens varies from 20 kg/m^3^ to 60 kg/m^3^, the water vapour resistance factor can be adopted: for SW specimens—μ = 2.537; for HF specimens—μ = 2.677; for SW/HF specimens—μ = 2.619. The variation analysis between different types of specimens shows that the averages of the water vapour resistance factor µ differ. The statistic of the F-criterion is 160, and *p* = 0. This indicates a statistically significant difference in the results of the subject. Consequently, the coefficient of determination R^2^ = 0.71 and the adjusted coefficient of determination R^2^ = 0.70 are obtained.

It can be seen that the highest difference is between SW- and HF-based thermal insulation materials, which is 5.5%. The difference between the SW- and SW/HF-based thermal insulation materials is 3.2%. The lowest difference in the water vapour resistance factor is obtained between thermal insulation materials made from HF and SW/HF, which is 2.3%. The difference in water vapour resistance factor values between SW, HF, and SW/HF thermal insulation materials can be explained by the similarity in fibre diameter. Since the difference in the thickness of the raw material of the fibres is very small, and the production process of the material itself is the same, the number of air gaps created between the fibres in the thermal insulation material, through which water vapour is transported, is also similar.

**Figure 7:**
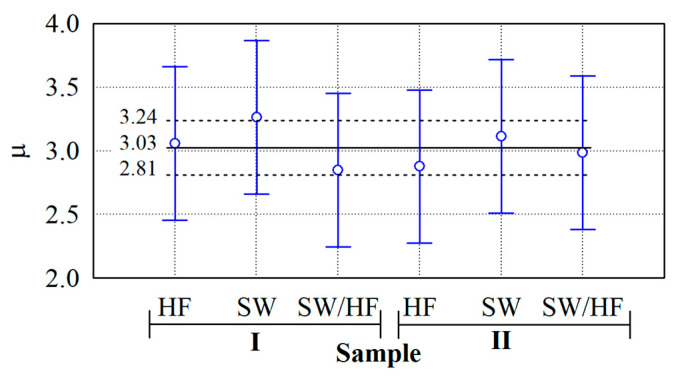
Test results of the water vapour resistance factor of thermal insulation materials coated with additives. Sample I—with hydrophobic agent; Sample II—with hydrophobic agent and flame retardant.

During repeated water vapour permeability tests, specimens with a 40 kg/m^3^ nominal density were selected. Furthermore, the specimens were coated solely with a hydrophobic agent and in conjunction with a flame retardant and a hydrophobic agent. The actual density values of specimens coated with a hydrophobic agent are as follows (see Figure 7, Sample I): SW—40.9 ± 0.493 kg/m^3^, HF—40.5 ± 0.265 kg/m^3^, and SW/HF—40.9 ± 0.173 kg/m^3^. The actual density values of specimens coated with a hydrophobic agent and a flame retardant are as follows (see Figure 7, Sample II): SW—41.1 ± 0.153 kg/m^3^, HF—40.9 ± 0.643 kg/m^3^, and SW/HF—40.9 ± 0.451 kg/m^3^.

Repeated analysis of water vapour permeability for specimens coated with various additives shows that the average values of the water vapour resistance factor µ do not differ (see Figure 7). The statistic of the F-criterion is 0.312, and *p* > 0.895. This indicates a statistically insignificant difference in the results of the subject. Consequently, the coefficient of determination R^2^ = (0.115) and the adjusted coefficient of determination R^2^ = (0.253) are obtained. The analysis performed shows that the relative vapour resistance factor can be taken as μ = 3.03 ± 0.428 for specimens (density 40 kg/m^3^) coated with a hydrophobic agent and specimens coated with both the hydrophobic agent and a flame retardant.

Other authors [48] have examined the water vapour permeability of SW-based mattresses. The products’ density ranged from 20 to 100 kg/m^3^, and the water vapour resistance factor from 2.6 to 9.7. In addition, the researchers used products with thicknesses varying from 6.5 to 180 mm. In the current case, the dependence of the water vapour resistance factor on the density has not been observed or the dependence is insignificant.

It is likely that in the work [49], the impact of the density is more evident due to the significant difference in thickness of the products analysed.

### 3.5. Flammability

For flammability assessment, the thermal insulation materials have been exposed to an open flame. The results of the flammability tests are presented in Table 7.

From the indicated data, it can be seen that the specimens of different uncoated thermal insulation materials are flammable, i.e., the flame spreads intensively on the surface of the samples. It can also be noted that the density and product orientation have no impact or a negligible impact on flame propagation.

To reduce the flammability of thermal insulation materials from natural resources, specimens of the selected density were coated solely with a flame retardant and with both a hydrophobic agent and a flame retardant (see Table 8). The data in Table 8 show that the flame does not reach the 150 mm standard limit in all cases. However, the flammability of the specimens coated solely with a flame retardant is lower than that of the specimens coated with both a hydrophobic agent and a flame retardant. It shows that the applied hydrophobic agent increases the flammability of all thermal insulation materials analysed, while the flame retardant increases the specimens’ wetness. Studies show that it is necessary to look out for more effective measures to reduce the flammability and water absorption of thermal insulation materials from natural fibres and evaluate the effectiveness of both additives when used in conjunction to increase the resistance to water and flame impact.

Flammability studies in the works of other authors are quite controversial. This is especially true in relation to SW. Scientists [49] point out that SW does not burn but rather carbonizes when exposed to a flame and does not contribute to the spread of the flame.

## 4. Conclusions

1. It is established that at the same density, not only does the thermal conductivity of thermal insulation materials made from different fibres differ but the variation in the intensity of thermal conductivity within the limits of different densities also differs. Using SW or SW/HF results in a lower thermal conductivity at lower densities than using HF.
2. Research on the tensile strength of thermal insulation materials from natural fibres shows that the tensile strength of the specimens prepared across the forming direction is approximately twice that of along-oriented specimens. Additionally, a two-fold higher density results in a two-fold increase in the tensile strength of thermal insulation materials with across-oriented fibres and a three-fold increase with along-oriented fibres.
3. It is established that the main factors determining the water absorption of thermal insulation materials from natural fibres are the product’s density, the nature of the fibres, and the use of a hydrophobic agent. The two-fold higher density of the product results in a two-fold increase in water absorption; the water absorption of products made from SW is approximately three-times lower than that of products made from HF. It is possible to reduce the water absorption by up to 75% by applying a hydrophobic agent.
4. It is established that the density from 20 to 60 kg/m^3^ of thermal insulation materials made from SW, HF, and SW/HF fibres does not impact the water vapour permeability values or the influence is negligible. The effect of the nature of the fibre on the water vapour permeability is also insignificant. The water vapour resistance factor of natural fibre-based thermal insulation materials of all studied compositions is within 5.5% or varies from 2.537 to 2.677.
5. All uncoated thermal insulation materials made of natural fibres are characterised by intense flammability, i.e., the fire spreads over the surface of the specimen. A flame retardant allows the fire to be inhibited from spreading on the surface of the specimen. In almost all cases, the flame height exceeds 100 mm. In addition, a hydrophobic agent increases the flammability of the resulting thermal insulation materials.

The results of this study expand the spectrum and data of research on the composition and properties of natural fibre-based thermal insulation materials and create additional opportunities to choose appropriate materials according to the appropriate needs. In future studies, more attention should be paid to the quantitative distribution of fibres and their diameters in the raw material as well as to the means of processing the surface of the fibres themselves, allowing effective protection against the effects of water and fire.

## Figures and Tables

**Table 1:** Compositions of thermal insulation materials.

Raw Materials, wt.%			
Composition	SW	HF	PLA^*)^
SW	100	0	20
HF	0	100	
SW/HF	50	50	

^*)^ The PLA content was calculated from the mass of SW and industrial HF.

**Table 2:** Results of statistical data for the thermal conductivity of the thermal insulation materials.

Composition	Specimens	Statistical Characteristics								
		$b_{0}$	$b_{1}$	$b_{2}$	R	R^2^	Adjusted R^2^	$S_{r}$	F	*p*
SW	27	$\lambda_{10{}^{\circ}C}=b_{0}+b_{1}\cdot \rho +b_{2}\cdot \rho^{2}$, (1)								
		0.05916	−0.0009406	0.000008560	0.982	0.964	0.961	0.000704	320.9	0
HF	27	$\lambda_{10{}^{\circ}C}=b_{0}+b_{1}\cdot \rho +b_{2}\cdot \rho^{2}$, (2)								
		0.04365	−0.000181	0.000001432	0.986	0.971	0.969	0.000162	405.2	0
SW/HF	27	$\lambda_{10{}^{\circ}C}=b_{0}+b_{1}\cdot \rho +b_{2}\cdot \rho^{2}$, (3)								
		0.04842	−0.0004385	0.000003642	0.990	0.981	0.979	0.000288	622.2	0

Note: $\rho \text{—}density,{kg/m}^{3};$ $\lambda_{10{}^{\circ}C}$—thermal conductivity, $\frac{W}{\left(m\cdot K\right)}$.

**Table 3:** Statistical results of the tensile strength measurement data of the thermal insulation materials.

Composition No.	Specimens	Statistical Characteristics							
		$b_{0}$	$b_{1}$	R	R^2^	Adjusted R^2^	$S_{r}$	F	*p*
SW	27	$\sigma_{tacross}=b_{0}+b_{1}\cdot \rho$, (4)							
		−6.11359	0.60381	0.991	0.982	0.981	1.109	1327	0
HF	27	$\sigma_{tacross}=b_{0}+b_{1}\cdot \rho$, (5)							
		−27.3799	2.51268	0.989	0.979	0.978	4.864	1210	0
SW/HF	27	$\sigma_{tacross}=b_{0}+b_{1}\cdot \rho$, (6)							
		−11.8468	1.47197	0.990	0.981	0.980	2.785	1270	0
SW	27	$\sigma_{talong}=b_{0}+b_{1}\cdot \rho$, (7)							
		−4.47562	0.33602	0.985	0.971	0.967	0.782	823	0
HF	27	$\sigma_{talong}=b_{0}+b_{1}\cdot \rho$, (8)							
		−18.5092	1.29079	0.992	0.984	0.983	2.219	1506	0
SW/HF	27	$\sigma_{talong}=b_{0}+b_{1}\cdot \rho$, (9)							
		−3.34429	0.49522	0.992	0.985	0.984	0.848	1605	0

Note: $\rho \text{—}density,{kg/m}^{3};$ $\sigma_{t}$—tensile strength, kPa.

**Table 4:** Statistical results of the short-term water absorption measurement data of the thermal insulation materials.

Composition	Number of Specimens	Statistical Characteristics							
		$b_{0}$	$b_{1}$	R	R^2^	Adjusted R^2^	$S_{r}$	F	*p*
SW	36	$W_{p}=b_{0}+b_{1}\cdot \rho$, (10)							
		−0.6595	0.05913	0.989	0.978	0.977	0.116	1546	0
HF	36	$W_{p}=b_{0}+b_{1}\cdot \rho$, (11)							
		−2.1544	0.17763	0.987	0.974	0.973	0.392	1265	0
SW/HF	36	$W_{p}=b_{0}+b_{1}\cdot \rho$, (12)							
		−1.6484	0.13478	0.989	0.977	0.976	0.269	1472	0

Note: $\rho —density,\;{kg/m}^{3};$ $W_{p}—$ short-term water absorption, kg/m^2^.

**Table 5:** The density results of the thermal insulation materials from the water vapour resistance factor determination test.

Composition	Indicator	Default Density kg/m^3^								
		20	25	30	35	40	45	50	55	60
SW	density	20.4	25.5	29.8	35.1	39.5	45.5	50.4	55.1	60.5
	standard deviation	0.305	0.336	0.439	0.114	0.488	0.383	0.396	0.152	0.460
HF	density	20.2	25.0	30.0	35.6	40.1	45.1	50.3	54.9	60.0
	standard deviation	0.647	0.487	0.757	0.329	0.552	0.344	0.636	0.421	0.513
SW/HF	density	20.0	25.1	30.0	35.1	39.8	45.2	50.3	54.9	59.9
	standard deviation	0.321	0.336	0.261	0.261	0.597	0.370	0.416	0.349	0.746

**Table 6:** Statistical analysis of the water vapour resistance factor of the thermal insulation materials.

Number of Specimens	Composition	Statistical Results					
		R	R^2^	Adjusted R^2^	$S_{r}$	F	*p*
45	SW	0.18	0.033	−0.18	0.027	0.16	0.99
45	HF	0.17	0.028	−0.19	0.049	0.13	0.99
45	SW/HF	0.22	0.049	−0.16	0.033	0.23	0.98
	All	0.84	0.71	0.70		160	0.00

**Table 7:** Assessment of the flammability parameters of uncoated thermal insulation materials.

Direction	Density, kg/m^3^			Combustion after Flame Is Extinguished			Flame Reaches 150 mm			Duration of Reaching 150 mm, s			Smoking			Filter Paper Ignition		
	SW	HF	SW/HF	SW	HF	SW/HF	SW	HF	SW/HF	SW	HF	SW/HF	SW	HF	SW/HF	SW	HF	SW/HF
→	19.8 ± 0.503	19.9 ± 0.802	19.7 ± 0.351	–	–	–	+	+	+	6 ± 2	6 ± 3	7 ± 2	–	–	–	–	–	–
↑	19.7 ± 0.651	20.1 ± 0.874	20.0 ± 0.794	–	–	–	+	+	+	8 ± 3	6 ± 1	5 ± 2	–	–	–	–	–	–
→	24.9 ± 0.603	25.1 ± 0.252	24.8 ± 0.265	–	–	–	+	+	+	7 ± 1	6 ± 2	8 ± 2	–	–	–	–	–	–
↑	25.5 ± 0.208	25.3 ± 0.600	25.1 ± 0.200	–	–	–	+	+	+	6 ± 0	6 ± 2	4 ± 1	–	–	–	–	–	–
→	30.0 ± 0.529	29.8 ± 0.889	30.1 ± 0.624	–	–	–	+	+	+	9 ± 3	4 ± 1	5 ± 3	–	–	–	–	–	–
↑	30.5 ± 0.611	30.6 ± 0.351	30.7 ± 0.473	–	–	–	+	+	+	6 ± 1	7 ± 3	5 ± 2	–	–	–	–	–	–
→	35.0 ± 0.200	35.5 ± 0.451	34.7 ± 0.503	–	–	–	+	+	+	7 ± 2	4 ± 2	8 ± 2	–	–	–	–	–	–
↑	35.3 ± 0.529	35.9 ± 0.321	35.7 ± 0.200	–	–	–	+	+	+	7 ± 3	6 ± 2	6 ± 3	–	–	–	–	–	–
→	39.7 ± 0.569	39.9 ± 0.651	39.6 ± 0.404	–	–	–	+	+	+	9 ± 2	8 ± 3	5 ± 3	–	–	–	–	–	–
↑	40.5 ± 0.964	41.0 ± 0.265	40.0 ± 0.300	–	–	–	+	+	+	8 ± 2	7 ± 1	6 ± 4	–	–	–	–	–	–
→	45.0 ± 0.651	45.0 ± 0.173	44.8 ± 0.436	–	–	–	+	+	+	9 ± 2	5 ± 1	7 ± 2	–	–	–	–	–	–
↑	45.4 ± 0.503	45.7 ± 0.361	45.3 ± 0.400	–	–	–	+	+	+	9 ± 2	7 ± 3	7 ± 1	–	–	–	–	–	–
→	50.0 ± 0.404	49.7 ± 0.436	49.4 ± 0.404	–	–	–	+	+	+	8 ± 3	7 ± 2	6 ± 3	–	–	–	–	–	–
↑	50.1 ± 0.872	50.7 ± 0.404	50.1 ± 0.265	–	–	–	+	+	+	7 ± 2	6 ± 4	9 ± 2	–	–	–	–	–	–
→	55.4 ± 0.404	55.0 ± 0.208	54.8 ± 0.473	–	–	–	+	+	+	9 ± 2	6 ± 3	8 ± 1	–	–	–	–	–	–
↑	55.2 ± 0.436	55.8 ± 0.436	54.8 ± 0.153	–	–	–	+	+	+	9 ± 3	8 ± 2	3 ± 2	–	–	–	–	–	–
→	60.1 ± 0.400	59.4 ± 0.451	60.3 ± 0.777	–	–	–	+	+	+	9 ± 1	8 ± 3	7 ± 2	–	–	–	–	–	–
↑	60.8 ± 0.200	60.9 ± 0.153	60.5 ± 0.503	–	–	–	+	+	+	10 ± 1	7 ± 3	6 ± 3	–	–	–	–	–	–

**Table 8:** Assessment of the flammability parameters of coated thermal insulation materials.

Direction	Density, kg/m^3^			Combustion after Flame Is Extinguished			Flame Reaches 150 mm			Duration of Reaching 150 mm, s			Smoking			Filter Paper Ignition		
	SW	HF	SW/HF	SW	HF	SW/HF	SW	HF	SW/HF	SW	HF	SW/HF	SW	HF	SW/HF	SW	HF	SW/HF
Coated with flame retardant																		
→	40.0 ± 0.100	40.0 ± 0.208	40.1 ± 0.153	–	–	–	125.0 ± 10.0	98.3 ± 7.63	105.0 ± 5.00	–	–	–	–	–	–	–	–	–
↑	40.1 ± 0.265	40.1 ± 0.200	40.9 ± 0.115	–	–	–	130 ± 5.00	105 ± 5.00	100.0 ± 17.3	–	–	–	–	–	–	–	–	–
Coated with hydrophobic agent and flame retardant																		
→	40.8 ± 0.404	40.7 ± 0.404	40.5 ± 0.252	–	–	–	136.7 ± 5.77	133.3 ± 2.89	135.0 ± 8.66	–	–	–	–	–	–	–	–	–
↑	40.6 ± 0.529	40.0 ± 0.100	40.9 ± 0.153	–	–	–	135.0 ± 5.00	130.0 ± 10.0	133.3 ± 11.55	–	–	–	–	–	–	–	–	–

## Data Availability

The original contributions presented in the study are included in the article, further inquiries can be directed to the corresponding author.
